# Lorlatinib in the second line and beyond for ALK positive lung cancer: real-world data from resource-constrained settings

**DOI:** 10.1038/s44276-024-00055-9

**Published:** 2024-05-01

**Authors:** Amit Kumar, Akhil Kapoor, Vanita Noronha, Vijay Patil, Nandini Menon, Ajay Kumar Singh, Amit Joshi, Amit Janu, Rajiv Kumar Kaushal, Trupti Pai, Anuradha Chougule, Omshree Shetty, Kumar Prabhash

**Affiliations:** 1 Department of Medical Oncology, Homi Bhabha Cancer Hospital and Research Centre(A Unit of Tata Memorial Centre, Mumbai), Muzaffarpur-, 842001 Bihar, India; 2 Department of Medical Oncology, Mahamana Pandit Madan Mohan Malviya Cancer Center and Homi Bhabha Cancer Hospital (A Unit of Tata Memorial Centre, Mumbai), Varanasi, 221005 Uttar Pradesh India; 3 Department of Medical Oncology, Tata Memorial Hospital, Tata Memorial Centre, Homi Bhabha National Institute, Mumbai, 400012 Maharashtra India; 4 Department of Radiology, Tata Memorial Hospital, Tata Memorial Centre, Homi Bhabha National Institute, Mumbai, 400012 Maharashtra India; 5 Department of Pathology, Tata Memorial Hospital, Tata Memorial Centre, Homi Bhabha National Institute, Mumbai, 400012 Maharashtra India; 6 Department of Molecular Pathology, Tata Memorial Hospital, Tata Memorial Centre, Homi Bhabha National Institute, Mumbai, 400012 Maharashtra India

## Abstract

**Background:**

ALK-positive lung cancers are known to have favorable responses with oral tyrosine kinase inhibitors. Lorlatinib is an approved treatment option post first and second-line ALK inhibitors and is now also in first line. We present a retrospective observational study of the safety and efficacy of patients receiving Lorlatinib in second-line and beyond.

**Methods:**

We conducted a retrospective observational study of ALK-positive patients who received Lorlatinib post-progression or intolerance to initial therapy at the Medical Oncology department. The patients who were started on Lorlatinib between January 2018 to December 2019 were included. The patients underwent routine blood and radiological evaluation every two to three months.

**Results:**

A total of 38 patients received Lorlatinib in the specified period. The median age was 48 years (range 23–68), with 53% of patients being male, 37% having comorbidities; the most common being hypertension and diabetes and 79% of patients were of ECOG-PS1. Twenty-two patients (58%) had received two prior TKIs. The most common sites of metastasis before starting Lorlatinib were brain (55%) and bone (53%). All patients except one received prior whole-brain radiotherapy with 4 receiving radiation twice. The median follow-up period was 49 months (95% CI: 46.4–51.6). Eighty-four percent showed disease control with median progression-free survival (PFS) and overall survival (OS) of 16 months (95% CI 5.4–26.6) and 22 months (95% CI 9.9–34.1) respectively. Twelve patients died without documented progression. Five out of twelve with documented progression had brain involvement while six had lung involvement. Twelve out of twenty-four patients who progressed received subsequent chemotherapy. The most common grade 3 and above toxicities were hypercholesterolemia and hypertriglyceridemia. Three (7.8%) patients required dose reduction.

**Conclusion:**

This real-world data confirms the efficacy of Lorlatinib in the second line and beyond with adverse effects matching that of registration studies.

## Introduction

The advent of targeted therapy has made significant improvements in the outcome of lung cancer in the past decade. Two decades back, it was discussed whether to treat the patient or give supportive care alone and today, we are discussing five-year survival outcomes with tyrosine kinase inhibitors (TKIs). After EGFR mutation, the second most frequent actionable mutation is ALK kinase domain rearrangement which is detected by immunohistochemistry, FISH, or NGS techniques in 3–5% followed by ROS-1 in 1–2% of patients [1]. The clinical presentation making ALK-positive patients different from other Non-small cell lung carcinoma were younger age, female sex predominance, never or light smokers, and a greater propensity for brain metastases [2]. The initial therapy with first-generation ALK inhibitor, Crizotinib provides a PFS of about 10.9 months in ALK-positive patients while the second-generation, ceritinib, and alectinib further improve PFS to 17–35 months [3–5]. The major drawback of first-generation drugs is poor brain penetration which leads to a great percentage of patients relapsing in the brain [6]. Though the drugs are usually well-tolerated, some patients may need to discontinue or modify the dose due to toxicities. The inherent nature of tumors gradually leads to the development of mutation causing resistance to these drugs [7, 8].

Lorlatinib is a third-generation, highly potent, macrocyclic ALK/ROS1 TKI that competitively binds to the adenosine triphosphate‐binding pocket, blocking ALK‐dependent oncogenic signaling. The advantage of Lorlatinib is high penetration of the blood-brain barrier by decreasing p‐glycoprotein‐1‐mediated efflux [9–11]. Besides, it has broad‐spectrum activity against most known resistance mutations that develop during treatment with first and second‐generation ALK TKIs, including *ALK G1202R* mutation [12]. The introduction of Lorlatinib to salvage these patients has shown the potential to add life. This has been shown in a global phase II study and other real-world studies, however, data is scant from LMIC [13–17]. The most common toxicities were peripheral edema (9–48%), hyperlipidemia (47–94%), weight gain (3–25%), peripheral neuropathy (30%), fatigue (15–30%) and cognitive effect (6–18%) in earlier studies. The treatment discontinuation rate varied from 3–14% due to toxicity [13, 16–18].

While Lorlatinib has emerged as a preferred treatment for ALK-positive lung cancer, its high cost often prevents many patients in low- to middle-income countries (LMICs) from accessing it. In these countries, patients primarily rely on self-funding for treatment, supplemented by support from non-governmental organizations (NGOs). Government funding for chemotherapy is limited, with targeted therapy and immunotherapy often not covered.

In a previous study, the authors present real-world data on the challenges faced in providing first-line treatment for ALK-positive lung cancer in LMICs [2]. ALK testing in these settings typically involves immunohistochemistry (IHC) or break-apart fluorescence in situ hybridization (FISH), although the use of limited panel next-generation sequencing (NGS) is on the rise due to cost reduction.

This study presents the safety and efficacy of patients receiving Lorlatinib in the second line and beyond for ALK- positive lung cancer in a resource-constrained settings.

## Patients and methods

This study is a retrospective audit of a prospectively collected database at the Department of Medical Oncology, Tata Memorial Hospital, Mumbai, India. The details of the patients were obtained from the prospective lung cancer audit database, wherein patients sign a written informed consent before their information is recorded as a part of the lung cancer audit. The lung cancer audit is an Institutional Ethics Committee (IEC) approved observational protocol, is registered with the Clinical Trials Registry India (registration number: CTRI/2013/01/003335). Other relevant clinical details were obtained from hospital Electronic Medical Records (EMR). The study was conducted according to ethical guidelines established by the Declaration of Helsinki and other guidelines like Good Clinical Practice Guidelines and those established by the Indian Council of Medical Research (ICMR). The status of the patient who were not following up was updated by making telephone calls to the patients. All patients who were started on Lorlatinib for ALK positive lung cancer before 31st December 2019 were included for this analysis. No patient was excluded from the analysis.

The demographic details, histology, prior treatment, clinical and radiological response, date of disease progression, date of death and toxicity data were collected. Adverse events were graded according to CTCAE version 5.0. Since this study is a retrospective collection of data of patients treated with standard institutional protocol, ethical clearance was not sought.

The patients who were on Crizotinib were switched to second-generation drugs post-progression on the first line or intolerant to the first line. Further few patients were switched to Lorlatinib in the second line. The patients who were started on Ceritinib and Alectinib on progression or intolerance were switched to Lorlatinib. The patients were regularly followed up at two to three monthly intervals or seen earlier, if clinically indicated and underwent routine blood and radiological evaluation. The response evaluation was done according to RECISTv 1.1.

### Statistics

The survival endpoints were PFS and OS. PFS was defined as the time (in months) from the start of Lorlatinib to radiological progression or death. OS was the time in months from the start of Lorlatinib to death due to any cause. The OS from the date of primary diagnosis until death from any cause was also calculated. Overall response rate was calculated as the percentage of patients with complete response and partial response out of the total evaluable patients. Disease control rate was calculated as the percentage of patients with complete response, partial response and stable disease out of the total evaluable patients.

The qualitative data were analyzed with the Pearson Chi square test, Fisher’s exact test, or Mann-Whitney test. Kaplan Meier analysis was done to calculate PFS and OS. Survival curve comparisons were performed using the log-rank test. Hazard ratios were calculated using univariate Cox-regression analysis. All data were analyzed with SPSS version 23 (IBM Corp, NY, US).

## Results

### Baseline characteristics

A total of 38 patients received Lorlatinib between January 2018 to December 2019. Table 1 shows the baseline characteristics of the patients. The median age was 48 years (range 23–68 years); out of which 53% (*n* = 20) were males. Thirty-seven percent (*n* = 14) of patients had comorbidities; the most common being hypertension (21%) and diabetes (16%). Only 11% of patients had a smoking history. Seventy-nine percent of patients were of ECOG-PS-1 and 8% were ECOG-PS > 2 at the time of the start of Lorlatinib. Twenty-two patients (57%) received two prior TKIs. A total of 32 patients (84%) received two or more prior lines of therapy. The most common sites of metastasis before starting Lorlatinib were brain (55%), bone (53%) and lymph node (50%). The next in sequence were liver (40%), lung (37%), pleural effusion (37%), pleural nodules (21%), adrenal (10%) and others (8%). All brain metastasis patients except one received prior whole-brain radiotherapy with 4 being irradiated twice. No patients were given stereotactic radiotherapy as all the patients who were given radiotherapy to brain had significant disease burden in brain.

**Table 1:** Baseline demographics (*n* = 38).

Median age (years) (range)	48 (23–68)
Sex	*n* (%)
Male	20 (52.6)
Female	18 (47.4)
Comorbidities (overlapping)	14 (36.8)
HTN	8 (21.0)
DM	6 (15.8)
Hypothyroidism	2 (5.3)
CAD	2 (5.3)
Rheumatic Heart Disease	2 (5.3)
COPD	1 (2.6)
Smoker *n* (%)	4 (10.5)
Previous lines of therapy	
1	5 (13.2)
2	17 (44.7)
3	9 (23.7)
>4	7 (18.4)
Crizotinib	32 (84.2)
>1 TKI	22 (57.9)
2^nd^ Gen TKI	29 (76.3)
ECOG PS	
1	30 (78.9)
2	5 (13.1)
>2	3 (7.9)
Site of Mets (Overlapping)	
Brain	21 (55.3)
Bone	20 (52.6)
Nodal	19 (50.0)
Liver	15 (39.5)
Lung	14 (36.8)
Pleural effusion	14 (36.8)
Pleural nodules	8 (21.1)
Adrenal	4 (10.5)
Others (Abdominal wall, muscle, breast)	3 (7.9)

*HTN* hypertension, *DM* diabetes mellitus, *COPD* chronic obstructive pulmonary disease, *CAD* Coronary artery disease, *TKI* Tyrosine kinase inhibitor, *ECOG-PS* Eastern Co-operative Oncology Group- Performance status.

### Response assessment

**Fig. 1:**
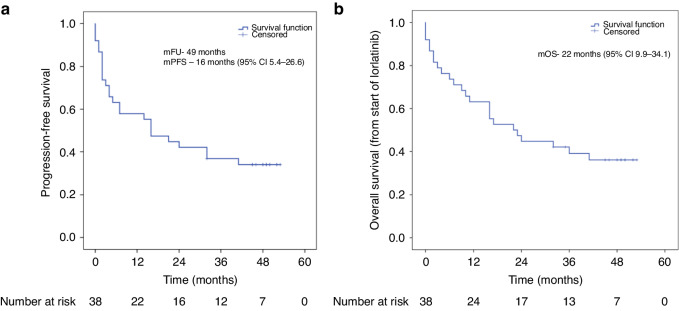
The median PFS and OS on Lorlatinib in second-line and beyond for ALK-positive lung cancers was 16 months (95% CI 5.4–26.6) and 22 months (95% CI 9.9–34.1), respectively. Kaplan–Meier survival curves depicting **a** progression-free survival and **b** overall survival on Lorlatinib.

**Fig. 2:**
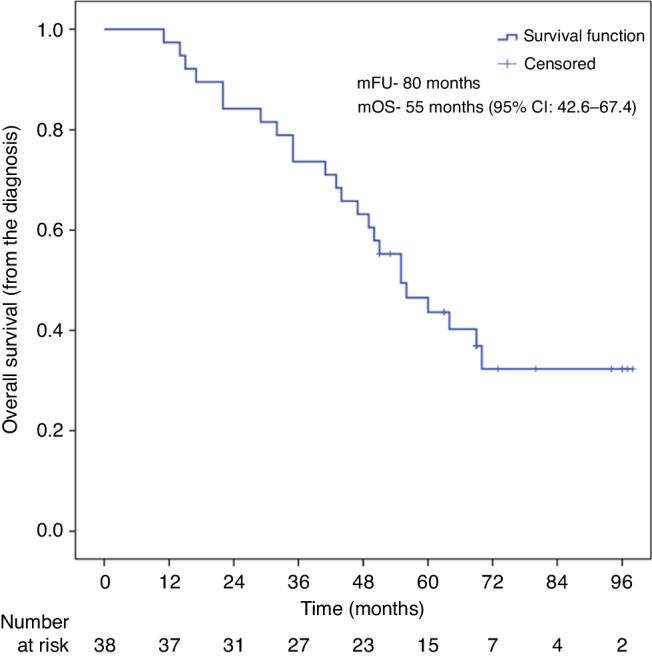
The median OS since diagnosis for ALK-positive lung cancer patients who received Lorlatinib as part of their treatment in second-line and beyond was 55 months (95% CI: 42.6–67.4). Kaplan–Meier survival curves depicting the overall survival of ALK-positive patients who received Lorlatinib.

The overall median follow-up duration was 80 months (95% CI: 62.9–97.0), and that on Lorlatinib was 49 months (95% CI: 46.4–51.6). Eighty-four percent showed disease control with median PFS and OS of 16 months (95% CI 5.4–26.6) and 22 months (95% CI 9.9–34.1) respectively (Fig. 1). The median OS since diagnosis was 55 months (95% CI: 42.6–67.4) (Fig. 2). One patient showed a complete response to Lorlatinib. Sixteen percent showed partial response while 65% had stable disease. Progression as the best response was seen in 16% of the patients. Seven patients were not evaluable radiologically. Table 2 shows the radiological responses of the patients. Twelve patients died without documented progression. Five out of twelve with documented progression had brain involvement while six had lung involvement. Twelve out of twenty-four patients who progressed received subsequent chemotherapy. Post-progression, Lorlatinib was continued in six patients with ablation of the site of the progression; two of which showed short-duration clinical response and one stable disease (Supplementary Tables 1 and 2).

**Table 2:** Best Response to therapy.

Best Response (*n* = 31)	*n* (%)
Progressive disease	5 (16.1)
Complete response	1 (3.2)
Partial response	5 (16.1)
Stable disease	20 (64.5)

Out of 38 patients, 7 patients were considered non-evaluable as scan could not be performed for them.

### Patients with brain metastasis at time of starting Lorlatinib

**Fig. 3:**
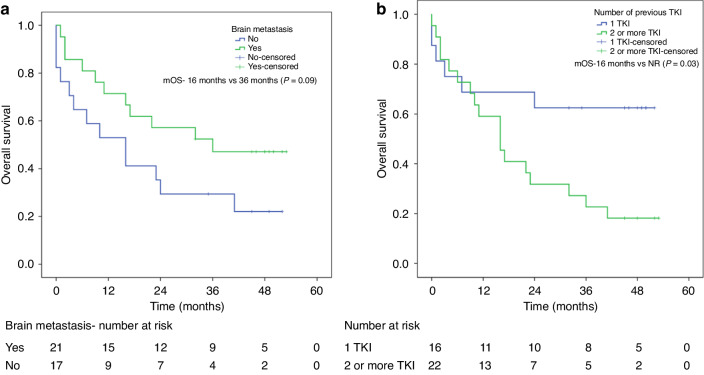
Kaplan–Meier survival curves depicting the overall survival of ALK-positive patients who received Lorlatinib with and without brain metastasis and those received one vs two or more TKI. **a** The median OS in patients with brain metastasis was 36 months (95% CI 23.4–41.5), which was statistically similar to patients without brain metastasis with a median OS of 16 months (95% CI 11.0–30.1) (*p* = 0.09). **b** The median OS of patients receiving more than one TKI and those receiving one TKI was 16 months vs NR (*p* = 0.03).

Out of 38 patients, 21 (55%) patients had brain metastasis at the time of starting Lorlatinib. The median PFS of patients with brain metastasis (*n* = 21) was 32 months (95% CI 20.4–39.2), which was statistically not different from the patients without brain metastasis (*n* = 17) with median PFS of 7.0 months (95% CI 0–17.5) (*p* = 0.061). The median OS in patients with brain metastasis was 36 months (95% CI 23.4–41.5), which was statistically similar to patients without brain metastasis with a median OS of 16 months (95% CI 11.0–30.1) (*p* = 0.09) (Fig. 3a).

### Previous TKI

Thirty-two patients (84%) received Crizotinib while 29 patients (76%) received 2nd generation TKI. Twenty-two patients (56%) received more than one TKI. The median PFS of patients receiving more than one TKI and those receiving one TKI was 7mth vs NR (*p* = 0.43). The median OS of patients receiving more than one TKI and those receiving one TKI was 16 months vs NR (*p* = 0.03) (Fig. 3b).

### Previous lines of therapy

**Fig. 4:**
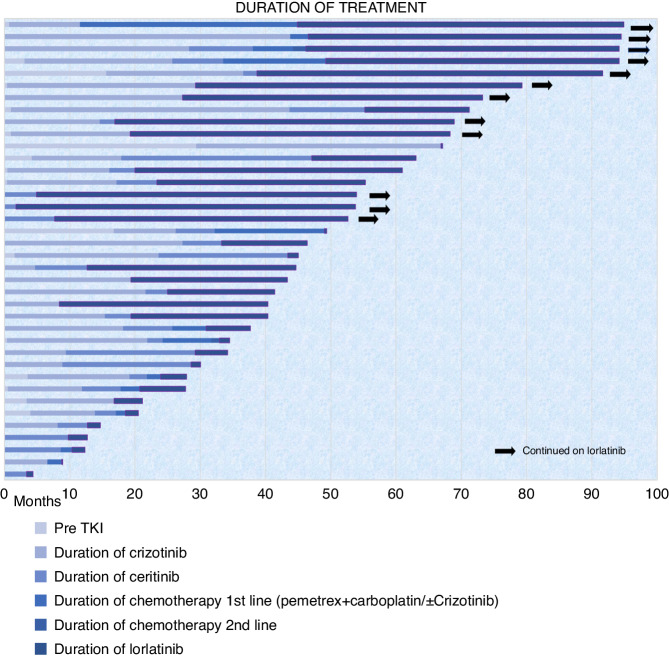
Swimmers plot showing the duration of therapy with different drugs.

Seven patients (18%) received four or more lines of therapy with median PFS of 4 months (95% CI 0–9.1) as compared to those with 1–3 lines of therapy having a median PFS of 21 months (95% CI 7.0–35.0) (*p* = 0.418). The median OS was 4 months (95% CI 0–9.1) and 24 months (95% CI 2.9–45.1) (*p* = 0.315) respectively. (Fig. 4)

### Adverse events

The most common grade 3 and above toxicity were hypercholesterolemia (13%) and hypertriglyceridemia (11%) whereas two patients developed anemia and one each hyponatremia, nausea, and hyperglycemia. Neurologic manifestation was seen in 6 (16%) patients. Three patients underwent a dose reduction to 75 mg due to anemia, delirium, and hallucination. It should be noted that 12 patients (32%) expired on treatment with Lorlatinib and this was ascertained to be due to disease progression. Supplementary Table 3 depicts the details of the adverse effects.

## Discussion

Apart from trial data, much real-world data on Lorlatinib has been reported from different regions of the world (Tables 3–5) [14–19]. This real-world evidence provides information on the course of disease and treatment outcomes in clinical settings, and thus insight into the generalizability of clinical trial findings in practice [20]. This real-world evidence is mainly derived from electronic health records, which are used for patient care rather than research. Therefore, the quality of this disorganized and unstructured dataset depends on the data curation process and on clinician and patient-related factors. This makes them different from controlled clinical trials [20]. The most common clinical endpoints are overall survival (OS), which is definite, but response rate (ORR) and progression-free survival (PFS) are based on clinical interpretation of imaging reports and symptomatic criteria [21].

**Table 3:** Demographic comparison of different Lorlatinib real-world studies and the trial data.

	Trial data (Solomon et al.)	EAP South Asia and US.	EAP Germany Frost et al.	EAP France. Baldacci et al.	Chinese data	Present study
Number	275	76	37	208	22	38
Median Age (years) (range)	54 (19–85)	53 (13–73)	58 (32–70)	60.9 (20.7–83.8)	49 (35–74)	47 (23–68)
Sex *n* (%)						
Male	118 (43)	33 (43)	19 (51.4)	91 (44)	10 (45.5)	22 (53.6)
Female	157 (57)	43 (57)	18 (48.6)	117 (56)	12 (54.5)	19 (46.4)
Current or former Smoker *n* (%)	NA	18 (23)	13 (35.1)	64 (31)	5 (22.7)	4 (9.6)
Previous lines of therapy-*n* (%)						
1	87 (38.6)	7 (9)	-	8 (4)	-	5 (12.2)
2	-	18 (24)	-	36 (17)	-	17 (41.5)
3	-	19 (25)	-	62 (30)	-	11(26.8)
>4	-	32 (42)	-	102 (49)	-	8 (19.5)
>1 TKI	111 (40.4)	66 (87)	34 (91.9)	188 (91)	12 (54.5)	24 (58.5)
2^nd^ Gen TKI	139 (50.5)	-	-	194 (93)	22 (100)	31 (75.6)
ECOG-PS-*n* (%)						
0–1	265 (96)		27 (73)	125 (72)	15 (68.2)	33 (80.5)
≥2	10 (4)		10 (27)	=>2- 48 (28)	7 (31.8)	8 (19.5)
Brain Mets	166 (60)	64 (84)	26 (70.3)	160 (77)	18 (75)	23 (59.0)

“EAP”- Expanded access program. “-” depicts data not available.

**Table 4:** Response comparison of different lorlatinib real world studies and the trial data.

	Trial data (Solomon et al.)	EAP South Asia and US. Zhu V et al. [18]	EAP Germany. Frost et al. [16]	EAP France. Baldacci et al. [17]	China. Lee et al. [19]	This study
Total number	229^*^	76	37	208	22	38
Number evaluable	215	64	14	191	22	31
mFU (months)	7.2	6.8	16.1	23.3	-	49
Best Response						
Progressive disease	34 (15.8)	13	-	25 (13)	5 (23)	5 (16)
Complete response	5 (2.3)	2	-	8 (4)	8 (36)	1 (3)
Partial response	115 (53.5)	19	-	85 (45)	5 (23)	5 (16)
Stable disease	60 (27.9)	30	-	71 (37)	4 (18)	20 (65)
mDOR (95% CI, months)	NR	-	10.4 (6.5-12.8)	14.9 (10.1 to NR)	-	-
ORR	51%	33%	42.4%	49%	35.7%	19%
DCR	79%	80%	86%	86%	64.3%	84%
mPFS (months)	NR	9.3	7.1	9.9	6.2	16
mOS (months)	NR	NR	24.7	32.9	NR	21
Brain metastasis		*N* = 9	*N* = 26	*N* = 160	*N* = 18	*N* = 21
mPFS (months)	NR	9.3	-	-	-	-
RR (Brain mets)	63%	35%	62.5% vs 35.7%	56%	100%	80%
Cause of treatment discontinuation 1. Disease progression 2. Toxicity 3. Death 4. Investigator’s decision 5. Patient ‘s decision 6. Intercurrent disease	-	-	-	60 (29) 28 (14) 15 (7) 7 (3) 1 (1) 1 (1)	-	12 (32) 0 (0) 12 (32) 0 0 0

“EAP”- Expanded access program. “-” depicts data not available.

*Only ALK positive patients included in this analysis (study involved both ALK and ROS positive patients).

**Table 5:** Adverse events of Lorlatinib in different studies.

	EAP South Asia and US. Zhu V et al. [18] *n* = 76		EAP Germany. Frost et al. [16] *n* = 37		EAP France. Baldacci et al. [17] *n* = 208	China. Lee et al. [19] *n* = 22		This study *n* = 38	
	G1–2 (%)	G3-5 (%)	G1 or 2 (%)	G3-5 (%)	G3-5 (%)	G1-2 (%)	G3-5 (%)	G1-2 (%)	G3-5 (%)
Any adverse event	-	-	37	10	62 (30)	-	-	-	-
Hypercholesterolemia	58 (61)	8 (8)	15 (29)	2 (4)	24 (12)	14 (74)*	4 (18.8)*	20 (53)	5 (13)
Hypertriglyceridemia	41 (43)	4 (4)	5 (10)	1 (2)	8 (4)	-	-	16 (42)	4 (11)
Edema	7 (7)	0	8 (16)	2 (4)	5 (2)	-	-	13 (34)	-
Cognitive disturbance	6 (6)	2 (2)	5 (10)	1 (2)	11 (5)	-	-	3 (8)	-
Dizziness	3 (3)	0	-	-	-	-	-	-	-
Weight gain	3 (3)	0	1 (2)	0	-	-	-	2 (5)	-
Hallucinations	2 (2)	1 (1)	-	-	-	-	-	3 (8)	-
Rash	2 (2)	0	1 (2)	0	-	-	-	-	-
Myalgia	2 (2)	0	1 (2)	0	-	-	-	1 (3)	-
Depression	2 (2)	0	-	-	-	-	-	-	-
ALT increased	1 (1)	1 (1)	-	-	-	-	-	2 (6)	-
Fatigue	1 (1)	0	2 (4)	0	3 (1)	-	-	4 (12)	-
Blood bilirubin increased	1 (1)	0	-	-	-	-	-	-	-
CPK increased	1 (1)	0	-	-	-	-	-	-	-
Hemorrhoids	1 (1)	0	-	-	-	-	-	-	-
Dry eye	1 (1)	0	-	-	-	-	-	-	-
Blurred vision	1 (1)	0	-	-	-	-	-	-	-
Peripheral neuropathy	1 (1)	0	2 (4)	0	5 (2)	-	-	3 (8)	-
Constipation	1 (1)	0	-	-	-	-	-	-	-
Pneumonitis	0	1 (1)	0	2 (4)	-	-	-	-	-
Gait disturbance	0	1 (1)	-	-	-	-	-	-	-
Diarrhea	-	-	3 (6)	0	-	-	-	3 (8)	-
Dysguesia	-	-	2 (4)	0	-	-	-	-	-
Lipase/Amylase increased	-	-	3 (6)	1 (2)	-	-	-	-	-
Mucositis	-	-	1 (2)	0	-	-	-	-	-
Nausea	-	-	1 (2)	0	-	-	-	4 (11)	-
Dyspnea	-	-	5 (10)	0	-	-	-	-	-
Pleural effusion	-	-	0	1 (2)	-	-	-	-	-
Pneumonia	-	-	2 (4)	0	-	-	-	2 (5)	1 (3)
Creatinine increased	-	-	1‘(2)	0	-	-	-	2 (5)	-
Hypertension	-	-	1 (2)	0	-	-	-	2 (5)	-
Hypothyroidism	-	-	2 (2)	0	-	-	-	4 (11)	-
Thromboembolism	-	-	1 (2)	1 (2)	-	-	-	-	-
Tongue swelling	-	-	0	0	-	-	-	-	-
Pruritus	-	-	1 (2)	0	-	-	-	-	-
Sweating	-	-	0	0	-	-	-	-	-
Ejection Fraction decrease	-	-	-	-	4 (2)	-	-	-	-
Mood effect	-	-	-	-	3 (1)	-	-	-	-
Arthralgia	-	-	-	-	2 (1)	-	-	2 (5)	-
Pulmonary hypertension	-	-	-	-	2 (1)	-	-	-	-
Anemia	-	-	-	-	-	-	-	11 (29)	2 (5)
Hyponatremia	-	-	-	-	-	-	-	4 (11)	1 (3)
Hyperglycemia	-	-	-	-	-	-	-	1 (3)	1 (3)
Hypokalemia	-	-	-	-	-	-	-	1 (3)	-
Hypophosphatemia	-	-	-	-	-	-	-	1 (3)	-
Hypomagnesemia	-	-	-	-	-	-	-	4 (11)	-
Hypoalbuminemia	-	-	-	-	-	-	-	4 (11)	-
Dyspepsia	-	-	-	-	-	-	-	3 (8)	-
Treatment Discontinuation due to toxicity	-	-	5	-	14 (28)	-	0	0	-

“EAP”- Expanded access program. “-” depicts data not available.

*Reported as dyslipidemia.

This data from a LMIC shows the efficacy and safety of Lorlatinib in ALK-positive patients after initial lines of TKIs. In India, a large number of ALK-positive patients still receive Crizotinib in the first-line settings. It should be noted that use of Crizotinib use is also feasible only after the support of non-governmental organizations (NGOs), in the absence of which a lot of patients will be devoid of any ALK-inhibitor during their course of disease. This has been discussed by the authors in more detail in another paper highlighting the practice patterns and clinical profile of ALK-positive lung cancer patients from India [2]. The ORR and DCR were 19% and 84%, respectively, for ALK-positive patients. These rates were comparable to those reported from the German Expanded Access Program (35% and 80%), French Expanded Access Program (49% and 87%), and other expanded access programs in Asian countries and the US (33% and 80%) [16–18]. The lower ORR as compared to other studies was due to the majority of patients having stable disease, as compared to partial or complete response. This is evident as DCR was similar across the studies. The median PFS was 16 months, as compared to around 7-11.2 months in previous studies [14–19]. This may be explained by the higher use of first-generation ALK TKI in first-line settings in our patients, and also a few patients switched to Lorlatinib because of intolerance to previous TKIs. The baseline characteristics and outcomes of different real-world studies are depicted in Tables 3 and 4.

The median OS since diagnosis was 55 months (95% CI: 42.6–67.4) in ALK-positive patients. This was lower than the other French real-world study, where patients receiving next-generation ALK inhibitors (mostly ceritinib and alectinib) had a median OS of 89.6 months. This was most likely due to selection bias in that study, where they excluded patients with poorer ECOG-PS, heavily pre-treated patients, and patients who had a poorer response to crizotinib. It could also be due to selection of patients with specific tumor biology and high sensitivity to ALK inhibition [22]. The OS in different expanded access programs was slightly better (24.7 months–not reached), but this could also be due to patient presentation in more advanced conditions in our setting [16–18].

In this study, 55% of patients had brain metastases before starting lorlatinib. This is similar to previous literature showing 60% of patients progressing in the brain over 3 years in ALK-positive cases [23]. There was no statistically significant difference in PFS and OS in patients with and without brain metastasis, pointing to its efficacy even in patients with brain metastases. One patient with prior brain metastasis who had not received radiotherapy continued on Lorlatinib for 41 months when she expired due to pneumonitis. The comparison between patients with and without brain metastasis is subject to various confounders, and it is not possible to draw conclusions regarding this data. Numerically there is a large difference in PFS between these two groups (32 months vs. 7 months) but it is not possible to draw a conclusion here given the heterogeneity of the data. Despite the intracranial activity of Lorlatinib, 5 out of 12 patients with documented progression on lorlatinib before death showed disease progression in the brain. So, a clear brain protective effect of lorlatinib could not be ascertained from our study.

The outcome was significantly better in patients who received one line of TKI than those who received 2 or more lines. The PFS and OS were not reached (NR) in patients exposed to one TKI, as compared to 7 months and 16 months, respectively, in those exposed to two or more TKIs. This trend was also seen in other real-world studies, where PFS were NR, 11.2 months, and 6.5 months in patients receiving two, two or more, and three or more TKIs, respectively [18]. In the French study LORLATU cohort, the PFS decreased from 11.7 months to 5.8 months depending on whether the patient had received two or more previous TKIs, suggesting earlier introduction of Lorlatinib showing benefit [17, 24, 25]. This has been further shown in the CROWN trial, where Lorlatinib had better PFS than crizotinib in the initial line, but a direct comparison with second-generation TKIs is lacking in trial settings. However, it should be noted that the PFS in later lines is always expected to be lesser than in earlier lines as patients in later lines are later in the disease course and ideally, OS difference is needed to establish the benefit of a specific drug sequencing.

The major grade 3 adverse events were dyslipidemia in 12% of patients, which is similar to the previously reported 15%. It was well managed with lipid-lowering agents and lifestyle modification. Similar data is seen from other real-world studies from Germany, France, China, the US, and other Asian countries (Table 5) [14–19].

The other most common toxicities were hypothyroidism, hyponatremia, anemia, and pedal edema. The incidence of hypothyroidism and anemia was slightly higher in our study than other real-world data. This may be due to the higher predisposition to anemia and thyroid disease in our population. Electrolyte imbalance was also seen in around 14% of patients. Though not severe, pedal edema was present in around 34% of patients. It required limb elevation, diuretics, and compression stockings for management. This is less than previous literature showing around 51% [9], but higher than other real-world data. Fatigue and diarrhea affected less than 10% of patients.

Only 3 patients (7%) required dose reduction due to anemia, hallucination, and neurologic toxicity, respectively. Two progressed within 2 and 6 months of starting lorlatinib, while one continued for 2 years and 8 months before progression. In this study, 18% of patients had neurologic toxicity, as compared to 23% reported in earlier studies. However, this is in line with data for incidence in the Asian population, which is around 12% as compared to non-Asian (28%). These included memory impairment, cognitive disorder, and amnesia, which usually presented within the first 2 months of treatment and were mainly grade ≤2 in severity. Peripheral neuropathy was seen in 8%, which was less than previously reported pooled data of 41% [9]. The reason for the same could not be ascertained, and could be related to the retrospective nature of the study. The weight gain was seen in only 5% of patients, which was comparable with real-world data but less than trial data. There was around 30% weight gain in affected patients [9]. A higher incidence of anemia and thyroid.

The major limitation of the study is its retrospective design. The patient cohort included patients with both disease progression and intolerance to previous drugs. Further, a few patients (*n* = 7) with advanced disease and heavily pre-treated died within 3 months of starting lorlatinib. We did not perform a biopsy prior to starting lorlatinib, and only 6 re-biopsies were done post-progression on lorlatinib. ALK kinase domain mutation testing was not performed due to resource constraints.

Lorlatinib has activity against most resistance mutations to first or second-generation ALK-TKIs, but its efficacy is reduced in the presence of an off-target resistance mechanism [26]. We were not able to analyze the effect of lorlatinib in different ALK fusion variants and their effect in comparison to chemotherapy in the case of an off-target mutation.

Another limitation was that no patient received immunotherapy either upfront or as subsequent lines post-progression on lorlatinib, mainly due to financial issues. However, an important strength of the study is the extended follow-up of the patients, with regular imaging done at 2–3 month intervals, and extensive documentation of toxicities.

## Conclusions

This real-world data from a resource-constrained settings demonstrates the effectiveness of Lorlatinib in patients who have progressed on first and second-generation TKIs and chemotherapy, and those who are intolerant to previous drugs. Although dyslipidemia and pedal edema are common side effects, they are usually manageable.

## Supplementary information

Supplementary Table 1

Supplementary Table 2

Supplementary Table 3

## Data Availability

The datasets generated during and/or analysed during the current study are available from the corresponding author on reasonable request.

## References

[1] Noronha V, Pinninti R, Patil VM, Joshi A, Prabhash K. Lung cancer in the Indian subcontinent. South Asian J Cancer. 2016;5:95–103. 27606290 10.4103/2278-330X.187571 PMC4991146

[2] Kapoor A, Noronha V, Patil V, Menon N, Joshi A, Kumar A, et al. Clinical profile, practice pattern, and outcomes with first-line therapy in ALK-positive lung cancer: real-world data from resource-constrained settings. JTO Clin Res Rep. 2022;4:100443. 36654881 10.1016/j.jtocrr.2022.100443 PMC9841022

[3] Solomon BJ, Mok T, Kim DW, Wu YL, Nakagawa K, Mekhail T, et al. First-line crizotinib versus chemotherapy in ALK-positive lung cancer. N. Engl. J. Med. 2014;371:2167–77. 25470694 10.1056/NEJMoa1408440

[4] Peters S, Camidge DR, Shaw AT, Gadgeel S, Ahn JS, Kim DW, et al. Alectinib versus crizotinib in untreated ALK-positive non- small-cell lung cancer. N Engl J Med. 2017;377:829–38. 28586279 10.1056/NEJMoa1704795

[5] Mok T, Camidge DR, Gadgeel SM, Rosell R, Dziadziusko R, Kim D-W, et al. Updated overall survival and final progression-free survival data for patients with treatment-naive advanced ALK-positive non-small-cell lung cancer in the ALEX study. Ann Oncol. 2020;31:1056–64. 32418886 10.1016/j.annonc.2020.04.478

[6] Gainor JF, Ou SH, Logan J, Borges LW, Shaw AT. The central nervous system as a sanctuary site in ALK-positive non-small-cell lung cancer. J Thorac Oncol. 2013;8:1570–3. 24389440 10.1097/JTO.0000000000000029

[7] Choi YL, Soda M, Yamashita Y, Ueno T, Takashima J, Nakajima T, et al. EML4-ALK mutations in lung cancer that confer resistance to ALK inhibitors. N Engl J Med. 2010;363:1734–9. 20979473 10.1056/NEJMoa1007478

[8] Katayama R, Shaw AT, Khan TM, Kenudson MM, Solomon BJ, Halmos B, et al. Mechanisms of acquired crizotinib resistance in ALK-rearranged lung cancers. Sci Transl Med. 2012;4:120ra17. 22277784 10.1126/scitranslmed.3003316 PMC3385512

[9] Bauer TM, Felip E, Solomon BJ, Thurm H, Peltz G, Chioda MD, et al. Clinical management of adverse events associated with Lorlatinib. Oncologist. 2019;24:1103–10. 30890623 10.1634/theoncologist.2018-0380 PMC6693708

[10] Johnson TW, Richardson PF, Bailey S, Brooun A, Burke BJ, Collins MR, et al. Discovery of (10R)‐7‐amino‐12‐fluoro‐2,10,16‐trimethyl‐15‐oxo‐10,15,16,17‐tetrahydro‐2h‐8,4‐(metheno)pyrazolo[4,3‐h][2,5,11]‐benzoxadiazacyclotetradecine‐3‐carbonitrile (PF‐06463922), a macrocyclic inhibitor of anaplastic lymphoma kinase (ALK) and c‐ros oncogene 1 (ROS1) with preclinical brain exposure and broad‐spectrum potency against ALK‐resistant mutations. J Med Chem. 2014;57:4720–44. 24819116 10.1021/jm500261q

[11] Schinkel AH. P‐glycoprotein, a gatekeeper in the blood‐brain barrier. Adv Drug Deliv Rev. 1999;36:179–94. 10837715 10.1016/s0169-409x(98)00085-4

[12] Zou HY, Friboulet L, Kodack DP, Engstrom LD, Li Q, West M, et al. PF‐06463922, an ALK/ROS1 inhibitor, overcomes resistance to first and second generation ALK inhibitors in preclinical models. Cancer Cell. 2015;28:70–81. 26144315 10.1016/j.ccell.2015.05.010 PMC4504786

[13] Solomon BJ, Besse B, Bauer TM, Felip E, Soo RA, Camidge DR, et al. Lorlatinib in patients with ALK-positive non-small-cell lung cancer: results from a global phase 2 study. Lancet Oncol. 2018;19:1654–67. 30413378 10.1016/S1470-2045(18)30649-1

[14] Peled N, Gillis R, Kilickap S, Froesch P, Orlov S, Flippova E, et al. GLASS: Global Lorlatinib for ALK(+) and ROS1(+) retrospective study: real world data of 123 NSCLC patients. Lung Cancer. 2020;148:48–54. 32799090 10.1016/j.lungcan.2020.07.022

[15] Soo RA, Huat-Tan E, Hayashi H, Seto T, Lin CC, Ou SHI, et al. Efficacy and safety of Lorlatinib in Asian and Non-Asian patients with ALK-positive advanced non-small cell lung cancer: subgroup analysis of a global phase 2 trial. Lung Cancer. 2022;169:67–76. 35660971 10.1016/j.lungcan.2022.05.012

[16] Frost N, Christopoulos P, Kauffmann-Guerrero D, Stratmann J, Riedel R, Schafaer M, et al. Lorlatinib in pretreated ALK- or ROS1-positive lung cancer and impact of TP53 co-mutations: results from the German early access program. Ther Adv Med Oncol. 2021;13:1758835920980558. 33613692 10.1177/1758835920980558 PMC7876585

[17] Baldacci S, Besse B, Avrillon V, Mennecier B, Mazieres J, Dubray-Longeras P, et al. Lorlatinib for advanced anaplastic lymphoma kinase–positive non–small cell lung cancer: results of the IFCT-1803 LORLATU cohort. Eur J Cancer. 2022;166:51–9. 35278825 10.1016/j.ejca.2022.01.018

[18] Zhu VW, Lin YT, Kim DW, Loong HH, Nagasaka M, To H, et al. An international real-world analysis of efficacy and safety of lorlatinib through early or expanded access programs in patients with TKI-refractory ALK+ or ROS1+ NSCLC. J Thorac Oncol. 2020;15:1484–96. 32360579 10.1016/j.jtho.2020.04.019

[19] Lee PH, Chen KC, Hsu KH, Huang YH, Tseng JH, Yang TY, et al. Real-world efficacy and safety of lorlatinib in treating advanced ALK-positive non–small cell lung cancer patients. Anti-Cancer Drugs. 2021;32:1099–104. 34232936 10.1097/CAD.0000000000001107

[20] Sherman RE, Anderson SA, Dal Pan GJ, Gray GW, Gross T, Hunter NL, et al. Real-world evidence—what is it and what can it tell us? N Engl J Med. 2016;375:2293–7. 27959688 10.1056/NEJMsb1609216

[21] Eisenhauer EA, Therasse P, Bogaerts J, Schwartz LH, Sargent D, Ford R, et al. New response evaluation criteria in solid tumours: revised RECIST guideline (version 1.1). Eur J Cancer. 2009;45:228–47. 19097774 10.1016/j.ejca.2008.10.026

[22] Duruisseaux M, Besse B, Cadranel J, Perol M, Mennecier B, Bigay-Game L, et al. Overall survival with crizotinib and next-generation ALK inhibitors in ALK-positive non-small-cell lung cancer (IFCT-1302 CLINALK): a French nationwide cohort retrospective study. Oncotarget. 2017;8:21903–17. 28423535 10.18632/oncotarget.15746 PMC5400633

[23] Rangachari D, Yamaguchi N, VanderLaan PA, Folch E, Mahadevan A, Floyd SR, et al. Brain metastases in patients with EGFR- mutated or ALK-rearranged non-small-cell lung cancers. Lung Cancer. 2015;88:108–11. 25682925 10.1016/j.lungcan.2015.01.020 PMC4355240

[24] Yoda S, Lin JJ, Lawrence MS, Burke BJ, Friboulet L, Langenbucher A, et al. Sequential ALK inhibitors can select for lorlatinib-resistant compound ALK mutations in ALK-positive lung cancer. Cancer Discov. 2018;8:714–29. 29650534 10.1158/2159-8290.CD-17-1256 PMC5984716

[25] Ou SH, Janne PA, Bartlett CH, Tang Y, Kim DW, Otterson GA, et al. Clinical benefit of continuing ALK inhibition with crizotinib beyond initial disease progression in patients with advanced ALK-positive NSCLC. Ann Oncol. 2014;25:415–22. 24478318 10.1093/annonc/mdt572

[26] Gainor JF, Dardaei L, Yoda S, Friboulet L, Leshchiner I, Katayama R, et al. Molecular mechanisms of resistance to first- and second-generation ALK inhibitors in ALK-rearranged lung cancer. Cancer Discov. 2016;6:1118–33. 27432227 10.1158/2159-8290.CD-16-0596 PMC5050111

