# Supplementary material for: Lorlatinib in the second line and beyond for ALK positive lung cancer: real-world data from resource-constrained settings
Source: BJC Rep. 2024 May 1;2:35. doi: 10.1038/s44276-024-00055-9 (PMC11523971; doi:10.1038/s44276-024-00055-9)
Supplement: Supplementary file 1 — Supplementary Table 1 [file 44276_2024_55_MOESM1_ESM.docx]

**Supplementary Table 1: Treatment post progression on Lorlatinib**

| Pemetrexed + Carboplatin + Bevacizumab | 6 |
| --- | --- |
| Single agent Gemcitabine | 1 |
| Docetaxel + Bevacizumab | 1 |
| Carboplatin + Etoposide | 1 |
| **Lorlatinib continued beyond progression** | 6 |
| SBRT to liver/lung | 2 |
| WBRT | 2 |
| SBRT: Stereotactic Body Radiotherapy; WBRT: Whole Brain Radiotherapy | |
