# Supplementary material for: Lorlatinib in the second line and beyond for ALK positive lung cancer: real-world data from resource-constrained settings
Source: BJC Rep. 2024 May 1;2:35. doi: 10.1038/s44276-024-00055-9 (PMC11523971; doi:10.1038/s44276-024-00055-9)
Supplement: Supplementary file 2 — Supplementary Table 2 [file 44276_2024_55_MOESM2_ESM.docx]

**Supplementary Table 2**: Site of progression (n=24)

| Death without documented progression | 12 |
| --- | --- |
| Documented progression | 12 |
| Brain | 5 |
| Lung | 6 |
| Pleural | 4 |
| Node | 3 |
| Bone | 3 |
