# Supplementary material for: Lorlatinib in the second line and beyond for ALK positive lung cancer: real-world data from resource-constrained settings
Source: BJC Rep. 2024 May 1;2:35. doi: 10.1038/s44276-024-00055-9 (PMC11523971; doi:10.1038/s44276-024-00055-9)
Supplement: Supplementary file 3 — Supplementary Table 3 [file 44276_2024_55_MOESM3_ESM.docx]

**Supplementary Table 3: Toxicities on treatment with Lorlatinib**

| **Toxicity n (%)** | **Grade 1** | **Grade 2** | **>Grade 2** |
| --- | --- | --- | --- |
| Hypercholesterolemia | 8 (21) | 12(32) | 5(13) |
| Hypertriglyceridemia | 10(26) | 6(16) | 4(11) |
| Hypothyroidism | 4(11) |  |  |
| Anemia | 7(18) | 4(11) | 2(5) |
| Hypoalbuminemia | 3(8) | 1(3) |  |
| Hyponatremia | 4(11) |  | 1(3) |
| Hypokalemia |  | 1(3) |  |
| Hypocalcemia | 2(5) |  |  |
| Hypophosphatemia | 1(3) |  |  |
| Hypomagnesemia | 4(11) |  |  |
| Transaminitis | 1(3) | 1(3) |  |
| Pedal edema | 12 (32) | 1(3) |  |
| Fatigue | 1(3) | 3(8) |  |
| Mouth dryness | 1(3) |  |  |
| Dyspepsia | 2(5) | 1(3) |  |
| Diarrhea | 1(3) | 2(5) |  |
| Arthritis |  | 2(5) |  |
| Myalgia |  | 1(3) |  |
| Nausea | 2(5) | 2(5) | 1(3) |
| Hallucination | 3(8) |  |  |
| Neurologic(Delirium) | 2(5) |  |  |
| Forgetfullness | 1(3) |  |  |
| HTN | 2(5) |  |  |
| Pneumonitis | 3(8) |  |  |
| Hyperglycemia |  | 1(3) | 1(3) |
| Renal dysfunction | 2 (5) |  |  |
| Peripheral neuropathy | 3 (8) |  |  |
| Weight gain |  | 2 (5) |  |
